# Neuromuscular and Neurocognitive Performance Associated with ACL Injury Risk in Youth Handball Players: A Prospective Cohort Study

**DOI:** 10.3390/sports14050185

**Published:** 2026-05-06

**Authors:** Gréta Csilla Sinka, Attila Pavlik, Ágnes Mayer, Dávid Fábián, András Pavlik, András Tállay

**Affiliations:** 1 Doctoral College, Semmelweis University, 1085 Budapest, Hungary; 2 Orthopaedic Surgery, Buda Health Centre, 1126 Budapest, Hungary; 3 Faculty of Sports Medicine, Semmelweis University, 1122 Budapest, Hungary; 4 Department of Physiotherapy, Faculty of Health Sciences, Semmelweis University, 1088 Budapest, Hungary; 5 National Institute for Sports Medicine, 1113 Budapest, Hungary

**Keywords:** neurocognition, ground contact time, handball, ImPACT, ACL

## Abstract

Background: Anterior cruciate ligament (ACL) injuries in youth athletes are multifactorial, and the relative contributions of neuromuscular and neurocognitive variables remain inadequately comprehended. Methods: In this prospective cohort study, 220 young handball players (104 girls and 116 boys; mean age 16.3 ± 1.4 years) participated in functional testing with the Back in Action system and baseline neurocognitive evaluation with the ImPACT battery. During the 24-month follow-up period, orthopedic specialists identified ACL damage, which was confirmed by magnetic resonance imaging (MRI). Univariable logistic regression and receiver operating characteristic (ROC) curve analyses were conducted to evaluate predictive capability. Results: During the 24-month follow-up, 26 athletes sustained an ACL injury. Prolonged plyometric ground contact time was significantly associated with ACL injury occurrence in logistic regression analysis (*p* = 0.019) and demonstrated fair discriminatory ability (AUC = 0.63) (OR = 0.98 per ms; 0.98 95% CI: 0.964–0.997). Female sex emerged as a profound and independent risk factor (OR = 5.74). Conclusions: Neuromuscular performance, specifically plyometric ground contact time and female sex, has predictive ability for ACL damage in youth handball players, while separate cognition assessments failed to independently differentiate injury risk. These findings support the use of objective neuromuscular evaluation in comprehensive injury prevention strategies in youth sport.

## 1. Introduction

Prior research has shown that, besides executing their own sport-specific motions, team players must also visually perceive the actions of their opponents and teammates [1,2,3,4]. Furthermore, team athletes have to react to stimuli as quickly as possible which can play a crucial role in the development of non-contact ACL injuries [5,6]. Delays in decision-making, attention, reaction time, or coordination due to time pressure can increase the risk of injury [7,8,9,10]. Consequently, it has been mentioned multiple times to not only assess physical performance but also to conduct sport-specific neurocognitive evaluations [10,11,12,13,14]. Bittencourt et al. were among the first to say that the etiology of injuries can only be properly examined with a complex approach; it is not enough to examine functional performance [2]. Neurological, biomechanical, neuromuscular, functional, and sport-specific factors contribute to a player’s injury [2]. Bahr and Krosshaug supplemented this by analyzing specific interactions between the athlete and their opponents [1]. Furthermore, physiological fatigue must be considered a critical component of this complex etiology, as it can significantly impair both cognitive processing and neuromuscular control [2,15,16,17,18,19]. Understanding the interplay between baseline neurocognitive skills and functional capacity is therefore essential to establish a criterion for injury predisposition before fatigue-induced performance degradation occurs. For example, in team sports such as handball, perception–action is key, meaning an athlete must be able to assess their own, their teammate’s, and the opponent’s actions in a certain situation under time pressure. The athlete also has to choose the best solution, as quickly as possible, and then implement it in a coordinated manner [5,6,15,16,17,18]. A possible solution can be a screening process which focuses on neurocognitive skills, such as visual–motor speed, reaction time, multitasking skills, visual memory, verbal memory, error rate, and the ability to make decisions [11,13,14,20,21,22,23,24].

Recent research suggests that injury etiology is a complex, multifactorial phenomenon where neurological, biomechanical, and neuromuscular factors converge [2]. Consequently, assessing functional performance in isolation may be insufficient to capture the full spectrum of injury risk; a comprehensive approach integrating neurocognitive evaluation is increasingly advocated [10,11,12,13,14]. One of the most validated tools for this purpose is ImPACT^®^ (Immediate Post-Concussion Assessment and Cognitive Testing) software, Version 4.0 (ImPACT Applications, Inc., Coralville, IA, USA), a computer-based system designed to measure domains such as visual–motor speed, reaction time, and memory. For instance, Herman et al. [8] found that athletes who subsequently sustained ACL injuries performed significantly worse in all ImPACT domains compared to healthy controls. This suggests that the neurocognitive deficits captured by ImPACT may manifest as a “cognitive–motor interference,” where the brain’s processing demands detract from the neuromuscular control required for joint stability.

To fully understand this interference, however, neurocognitive data must be integrated with objective measures of physical execution. While the ImPACT system assesses the “central” processing speed, functional deficits in strength, neuromuscular control, and dynamic balance—particularly lower-limb asymmetries—represent the “peripheral” risk factors that increase knee valgus loads [23,24]. To bridge this gap, the Back in Action (BIA) test battery (Corehab S.r.l., Trento, Italy) has been developed as a comprehensive functional screening tool. The BIA battery assesses agility, balance, and plyometric control through a series of increasingly complex tasks [25]. By utilizing both the ImPACT and BIA systems, it is possible to screen for a broader spectrum of risks, from delayed decision-making to the subsequent failure in neuromuscular compensation during high-demand sports like handball.

Despite the clinical relevance of these tools, there is currently no unified framework to analyze how position-specific neurocognitive and functional profiles correlate with ACL injury incidence. This study aims to address this gap by employing the ImPACT system for neurocognitive profiling and the Corehab BIA system for functional assessment.

The primary objective was to detect both the neurocognitive and functional profiles of elite players by gender and position, and to examine the correlation of these multidimensional profiles with ACL injury incidence. We hypothesized that: (1) suboptimal neuromuscular outcomes and functional asymmetries identified by the BIA test battery would correlate with a higher incidence of ACL injuries; and (2) diminished neurocognitive performance, specifically prolonged reaction time and reduced visual–motor speed measured with ImPACT, would be associated with increased ACL injury incidence.

## 2. Materials and Methods

### 2.1. Setting

This prospective cohort study included a baseline cross-sectional performance assessment followed by injury surveillance. A total of 220 youth handball players competing in the Hungarian National First League (U15–U20) were recruited between September and October 2023. Inclusion criteria were active roster status and full participation in team training. Athletes with an ACL injury within the previous 12 months were excluded. All participants and their legal guardians provided signed informed consent. The study was approved by the institutional ethics committee. The research license number from the Department of Clinical Research of the National Public Health and Pharmaceutical Center was NNGYK/03180-6/2025. Athletes were recruited from National First League youth teams. The measurements took place in Budapest, with a total of 220 people 16.33 ± 1.38 (104 girls 16.32 ± 1.39 and 116 16.34 ± 1.39 boys). Functional testing was done with the Back in Action (Corehab) system, while neurocognitive performance was tested with the ImPACT system. The athletes were then followed up for 24 months to record the ACL injury incidence. The injuries were reported by a doctor working alongside the teams.

Handball players who had an ACL injury in the last 1 year were excluded, and during the examined period the players who were medically diagnosed with an ACL injury were considered. ACL injury was defined as a complete or partial rupture confirmed by clinical examination and magnetic resonance imaging (MRI) and diagnosed by an orthopedic specialist.

### 2.2. Neuromuscular Performance—Back in Action System

Functional lower-limb performance was evaluated using the Back in Action (BIA) test battery (Corehab S.r.l., Trento, Italy), assessing postural stability, plyometric performance, agility, speed, and limb symmetry indices. The BIA testing battery utilizes its own proprietary stabilometer. The ‘Pass/Fail’ designation is determined by a proprietary algorithm that benchmarks the athlete’s performance against an internal database, adjusted for individual anthropometric variables (age, height, body mass, and BMI). This automated categorization eliminates potential subjective bias, as the investigator cannot manually influence or override the system-generated outcomes.

Table 1 shows the functional skills and their description. The test reliability is ICCs = 0.688–0.921 (depending on the subtest) [25]. The detailed description and photos of the subtests can be found in the Supplementary Materials.

### 2.3. Neurocognitive Performance—ImPACT System

Neurocognitive performance was assessed using the ImPACT^®^ (Immediate Post-Concussion Assessment and Cognitive Testing) software, Version 4.0 (ImPACT Applications, Inc., Coralville, IA, USA), which evaluates verbal memory, visual memory, visual–motor speed, reaction time, and dismiss. Testing was performed under standardized environmental conditions [10]. Regarding the ImPACT protocol: Participants did not perform warm-up trials to avoid practice effects, as per the standard clinical administration guidelines. Each specific task within the ImPACT battery consisted of three automated trials, with randomized stimuli to ensure data validity. To clarify the criteria for a ‘Pass’ result, we followed the international ImPACT standardization protocol. A ‘Pass’ was defined as achieving performance levels that met or exceeded the normative mean for the respective age group across the test modules. Specifically, a success rate of over 80% within the specific cognitive domains was required to satisfy the criteria.

The test reliability depends on the subtest components. Verbal memory ICC = 0.61; visual memory ICC = 0.68; reaction time ICC = 0.59; visual–motor speed ICC = 0.84. The composite scores and measurement details are included in the Supplementary Materials. Table 2 shows the subtests of each composite skill.

### 2.4. Testing

The neurocognitive test part was completed by the athletes on a weekend day at FTC Handball Academy in calm conditions. The regulations were complied with the cooperation and help of the parents. The test started at 10 am. Participants were instructed to refrain from electronic device use after 10:00 pm the night before testing and to get at least 8 h of sleep. To mitigate potential learning effects and task adaptation, participants were prohibited from performing any warm-up or practice sessions prior to the administration of the test.

The neuromuscular testing was held in the gym section of the academy, where the players arrived in teams, each of them on separate days. In 6 days, 6 teams were assessed, arriving from 10 am each day. On the day before the measurement, they were not allowed to participate in match situation training. On the day of the measurement, they were not allowed to participate in any kind of training.

### 2.5. Statistical Analysis

Statistical analyses were performed using IBM SPSS Statistics version 24.0 (IBM Corp., Armonk, NY, USA). Descriptive statistics (mean ± standard deviation or median and interquartile range, depending on distribution) were used to characterize the study population. Data normality was assessed using the Shapiro–Wilk test. To identify potential risk factors for ACL injury, univariable logistic regression analyses were first conducted for each neuromuscular and neurocognitive variable.

To address potential confounding factors as suggested during the peer-review process, sex was included as a covariate in our predictive models. The association between baseline neuromuscular, neurocognitive variables (e.g., ImPACT composite scores, 1-leg stability), and ACL injury occurrence was evaluated using multivariable logistic regression analysis. Results are reported as regression coefficients (ß), odds ratios (ORs), and 95% Confidence Intervals (CIs).

The discriminatory ability of the significant predictors was determined through receiver operating characteristic (ROC) curve analysis. The Area Under the Curve (AUC) was calculated to evaluate the overall model performance, and the optimal diagnostic threshold was identified using the Youden Index. Statistical significance was set at *p* ≤ 0.05 for all analyses.

## 3. Results

The mean age of the male participants (n = 116) was 16.34 ± 1.39 years, with an average body weight of 74.21 ± 12.45 kg, height of 182.39 ± 10.32 cm, and BMI of 22.40 kg/m^2^. Female participants (n = 104) had a mean age of 16.32 ± 1.40 years, body weight of 63.62 ± 10.27 kg, height of 170.85 ± 7.43 cm, and BMI of 21.75 kg/m^2^.

### 3.1. Neurocognitive Performance

Regarding neurocognitive screening, 26.9% of girls and 24.1% of boys passed the baseline tests while the majority (73.1% of girls and 75.9% of boys) did not reach the expected performance level.

Table 3 shows the results for verbal memory:

Table 4 shows the results for visual memory:

Table 5 shows the results for visual–motor speed:

Table 6 shows the results for reaction time:

Table 7 shows the results for dismiss:

Comparison of the performance in neurocognitive skills by gender and position is summarized in Table 8.

### 3.2. Functional Performance

In the functional tests, 80.8% of girls and 37.9% of boys met the passing criteria.

Table 9 shows the results for stability:

Table 10 shows the results for plyometrics and speed:

Table 11 shows the results for agility and obstacle course (Parkour):

Comparison of the neuromuscular performance by gender and position is summarized in Table 12.

### 3.3. Correlations with ACL Injury Incidence

In the examined period, there was a total of 26 ACL injuries. Exploratory correlation analyses were performed to screen potential associations between baseline variables and subsequent ACL injury occurrence. Moderate correlations were observed for reaction time and visual memory; however, these associations were not retained in logistic regression models. In contrast, plyometric ground contact time demonstrated consistent association patterns and was therefore further evaluated using predictive modeling.

### 3.4. Injury Prediction

#### Univariable Logistic Regression

Neurocognitive Predictors: The univariable models revealed that none of the baseline neurocognitive composite scores were significant independent predictors of subsequent ACL injury. Verbal memory (*p* > 0.05, AUC = 0.52) and reaction time (*p* > 0.05, AUC = 0.55) showed no significant association with injury risk. Visual memory performance similarly did not reach the level of statistical significance (*p* > 0.05, AUC = 0.54). Visual–motor speed and dismiss scores also demonstrated poor discriminatory capacity, with all AUC values falling between 0.50 and 0.55.

Neuromuscular Predictors: In contrast to the neurocognitive measures, plyometric performance showed a significant relationship with injury incidence. Plyometric ground contact time was a significant predictor of ACL injury (ß = −0.019, *p* = 0.019). The calculated odds ratio (OR) was 0.98 per millisecond (95% CI: 0.964–0.997), indicating that shorter ground contact times during plyometric tasks are associated with an increased probability of ACL injury. Other neuromuscular variables, such as quick-feet performance and stability scores, did not show significant independent predictive value (*p* > 0.05). Static Stability: Neither bilateral nor unilateral (dominant and non-dominant) stability scores were significantly associated with ACL injury occurrence (all *p* > 0.05). Two-leg stability showed poor discriminatory ability (AUC = 0.51). For unilateral stability, neither the dominant side (*p* = 0.42, AUC = 0.52) nor the non-dominant side (*p* = 0.38, AUC = 0.54) reached statistical significance. Symmetry index was not a significant independent predictor of injury (*p* > 0.05, AUC = 0.50). Quick-feet performance also failed to show a significant predictive relationship with subsequent injury (all *p* > 0.05).

**Figure 1:**
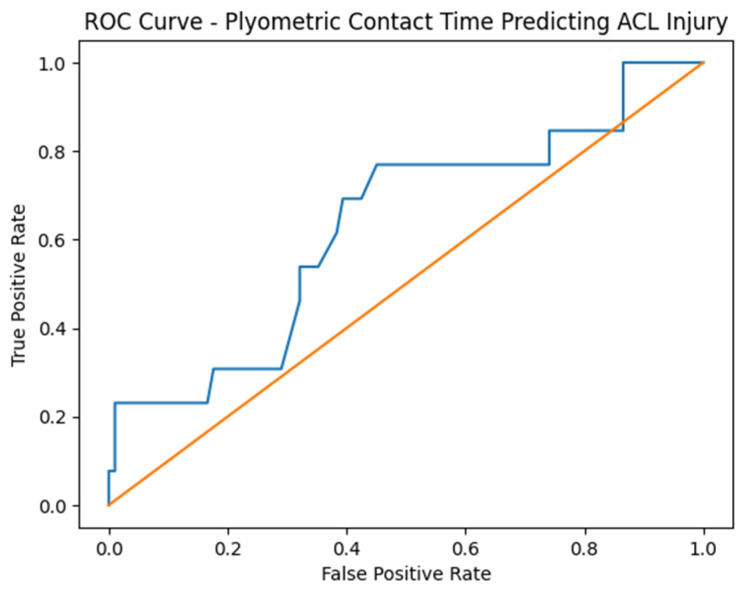
Plyometric contact time predicting ACL injury. The blue line represents the sensitivity and 1-specificity of the plyometric ground contact time model (AUC = 0.63). This value indicates a fair discriminatory capacity for identifying athletes at risk of ACL injury. The orange diagonal line serves as the reference line, representing chance-level prediction with an AUC of 0.50. Based on the Youden index, the optimal diagnostic threshold was identified at 168 ms. At this cutoff point, the model demonstrated a sensitivity of 77% and a specificity of 55% in predicting injury occurrence.

The final univariable model, predicting ACL injury from plyometric ground contact time, showed fair discriminatory capacity with an AUC of 0.63 (Figure 1). The optimal threshold determined by the Youden index was 168 ms, yielding a sensitivity of 77% and a specificity of 55%.

### 3.5. Multivariable Logistic Regression

To evaluate the independent predictive value of the examined parameters and to control for potential confounding, sex was included as a covariate in the multivariable models. In the combined model including both sex and plyometric ground contact time, sex emerged as the only significant independent predictor of ACL injury (*p* = 0.003, OR = 5.74, 95% CI: 1.78–18.48). This indicates that female athletes in this cohort were more than five times as likely to sustain an ACL injury compared to their male counterparts, regardless of other baseline measures.

Notably, when adjusted for sex, the independent effect of plyometric ground contact time was reduced and did not reach statistical significance (*p* = 0.35, OR = 0.99, 95% CI: 0.97–1.01). This suggests that the relationship between contact time and injury risk observed in the univariable model is significantly influenced by sex-specific differences in jump and landing mechanics. The overall discriminatory performance of the multivariable model yielded an AUC of 0.73.

## 4. Discussion

The primary finding of this prospective cohort study is that neuromuscular performance—particularly shortened plyometric ground contact time—was associated with subsequent ACL injury occurrence and demonstrated fair discriminatory capacity. Our findings challenge the independent predictive capacity of baseline neurocognitive screening in this specific cohort, while highlighting the critical interplay between plyometric landing mechanics and the athlete’s sex.

Ground contact time during plyometric tasks likely reflects impaired reactive force production and delayed neuromuscular activation strategies. Prior biomechanical studies show that non-contact ACL injuries generally occur within the initial 40 ms following ground contact, a timeframe marked by rapid force absorption and the necessity for joint stabilization [15,24]. Insufficient neuromuscular stiffness regulation and delayed eccentric control during this critical window may increase anterior tibial translation and valgus loading, thereby elevating ACL strain.

### 4.1. The Role of Neurocognitive and Stabilometric Performance

Contrary to our initial hypothesis and some existing literature [10,12], baseline neurocognitive scores (verbal/visual memory, reaction time, and processing speed) did not independently predict subsequent ACL injuries. Similarly, static and unilateral stabilometric measures failed to show significant predictive capacity.

A possible explanation is that while cognitive processing delays might contribute to injury mechanisms in chaotic, open-skill game situations, isolated baseline computer-based neurocognitive tests may lack the ecological validity to capture the dynamic, sport-specific decision-making processes required on the field. Furthermore, the lack of predictive value in static stabilometry suggests that dynamic, high-force tasks are more representative of the actual injury mechanism than static equilibrium.

### 4.2. Plyometric Ground Contact Time Landing Mechanics

In the univariable analysis, shorter plyometric ground contact time emerged as a significant predictor of ACL injury. Specifically, every 1 ms decrease in contact time increased the injury odds by approximately 2% (OR = 0.98).

From a biomechanical perspective, a shorter ground contact time during plyometric tasks often indicates a “stiff landing” strategy. Athletes adopting this strategy tend to absorb kinetic energy over a shorter duration, which typically involves reduced knee and hip flexion angles upon impact [14,18]. This limited energy attenuation through the lower extremity musculature exponentially increases the ground reaction forces and the sheer stress transmitted directly to the passive restraints of the knee, primarily the ACL.

### 4.3. The Confounding Influence of Sex

When adjusted for sex in the multivariable model, the independent predictive value of plyometric contact time diminished, while the female sex emerged as a profound and independent risk factor (OR = 5.74).

This shift implies a strong interaction between sex and neuromuscular landing strategies. Female athletes inherently demonstrate distinct anatomical (e.g., wider pelvis, increased Q-angle) and hormonal profiles, but crucially, they also tend to exhibit different neuromuscular control strategies, often landing with greater knee valgus and stiffer mechanics compared to males [22,25,26,27,28,29]. The multivariable model confirms that the high injury rate associated with shorter plyometric contact times is largely driven by these sex-specific biomechanical disparities.

### 4.4. Clinical and Rehabilitation Implications

For sports physiotherapists and rehabilitation specialists, these findings emphasize that injury prevention programs must prioritize dynamic neuromuscular training over static balance or purely cognitive tasks.

Since sex is a non-modifiable risk factor, clinical interventions must target the modifiable biomechanical components observed in female athletes. Training protocols should heavily emphasize the elongation of ground contact phases during deceleration and landing.

### 4.5. Limitations

Numerous limitations must be acknowledged. Initially, while the prospective design enhances causal inference, the limited number of ACL injury cases (n = 26) constrains the intricacy of multivariable modeling and may have reduced statistical power for identifying lower effect sizes. Secondly, neurocognitive performance was evaluated by standardized laboratory testing, which may not adequately reflect the dynamic perceptual–motor requirements seen in high-velocity, sport-specific contexts. Third, only baseline tests were incorporated, and any alterations in neuromuscular or cognitive performance during the follow-up period were not observed.

Additionally, external load exposure (such as training amount and match participation) and prior injury history were not accounted for, potentially affecting injury risk. Consequently, the current data must be understood within the framework of a multifactorial damage model rather than as proof of single-variable causation.

Future studies incorporating longitudinal neuromuscular monitoring and exposure-adjusted injury incidence may further clarify the interaction between cognitive processing and mechanical load in ACL injury mechanisms.

## 5. Conclusions

In this prospective cohort of youth handball players, neuromuscular performance—specifically plyometric ground contact time—demonstrated fair capacity to discriminate athletes at increased ACL injury risk, whereas isolated neurocognitive measures did not independently predict injury occurrence.

These findings reinforce the concept that ACL injury risk emerges from complex neuromechanical interactions and underscore the value of integrating objective neuromuscular assessment into comprehensive injury risk management strategies in youth sport.

### Future Directions

Future research should extend these findings to adult elite handball players to determine whether neuromuscular and neurocognitive profiles differ across developmental stages and competitive levels. Longitudinal monitoring over multiple seasons, combined with exposure-adjusted injury incidence, may provide a more precise understanding of temporal changes in neuromuscular performance and their relationship with ACL injury mechanisms.

Notably, most prior investigations have focused on adult elite populations; therefore, the present study extends the existing literature by providing prospective neuromuscular profiling data in youth handball athletes. This may facilitate age-appropriate screening and tailored development tactics inside talent development systems. Accordingly, neuromuscular assessment should be interpreted as a complementary component within a multifactorial injury prevention framework rather than as a standalone predictive tool.

## Figures and Tables

**Table 1:** Functional skills (BIA) and their detailed description.

Skill	Description
Stability	The test series begins with a two-leg stability test on a stabilometer with a trial attempt and three examinations. Then, there is an assessment of one-legged stability, also with a trial and two measurements. The players had to keep the ball (which represents the center of pressure) in the center of the target board marked on the laptop screen for 20 s.
Plyometrics	Four consecutive jumps were performed, during which the players have to jump as high as possible, with the least amount of time spent on the ground. Similar to stability tests, the plyometric jump is tested with one trial and two measurements, where the system evaluates both the height of the jumps and the ground contact time.
Speed	The speed was tested with the quick-feet test, where the players had to perform 15 stepping movements as quickly as possible on the stepladder manufactured for this purpose. There was a trial following with two measurements.
Agility	The Parkour (obstacle course) system examines knee joint stability in different directions, and dynamic balance. The player at the red step has to jump forward–backward–forward as fast as possible while standing on one leg, and at each blue step jump sideways. Here, after one trial, there are two measurements for both the dominant and non-dominant side.

**Table 2:** Neurocognitive skills (ImPACT) and their detailed description.

Skill	Description
Verbal memory	Evaluates attentional processes, learning, and memory within the verbal domain. This composite score represents the average performance on: Word Memory (Module 1) Total Percent Correct Symbol Match (Module 4) Total Correct Hidden/9 × 100 Three Letters (Module 6) Percent Total Letters Correct
Visual memory	Evaluates visual attention and scanning, learning, and memory. This score in its current form comprises the average of: Design Memory (Module 2) Total Percent Correct X’s and O’s (Module 3) (Total Correct Memory)/12 × 100
Visual–motor speed	Evaluates visual processing, learning and memory, and visual–motor response speed. This score comprises the average of the following scores: Total Number Correct/4 during Interference of X’s and O’s (Module 3). Average Counted Correctly × 3 from Countdown Phase of Three Letters (Module 6).
Reaction time	Evaluates average response speed. This score comprises the average of the following scores: Average Correct RT of Interference Stage of X’s and O’s (Module 3). Symbol Match (Module 4) Average Correct RT Visible/3. Color Match (Module 5) Average Correct RT.
Dismiss	Provides a measure of mistakes during each test module. This score is obtained by adding: Total Incorrect on the Interference Phase of X’s and O’s (Module 3). Color Match Total Commissions (Module 5).

**Table 3:** Verbal memory results per position.

	Average	Passed	Failed	Success %
Wingers	84.8 ± 10.1%	46 people	18 people	71.9%
Pivots	84.0 ± 9.0%	20 people	10 people	66.7%
Back players	83.7 ± 10.2%	38 people	22 people	63.3%
Goalkeepers	81.4 ± 8.9%	18 people	14 people	56.3%
Center backs	79.3 ± 13.9%	20 people	14 people	68.8%

**Table 4:** Visual memory results per position.

	Average	Passed	Failed	Success %
Wingers	75.2 ± 9.9%	29 people	3 people	90.6%
Pivots	73.6 ± 10.4%	24 people	6 people	80.0%
Back players	72.0 ± 14.3%	10 people	5 people	66.7%
Goalkeepers	71.7 ± 14.8%	12 people	4 people	75.0%
Center backs	70.6 ± 8.5%	14 people	3 people	82.4%

**Table 5:** Visual–motor speed results per position.

	Average	Passed	Failed	Success %
Center backs	37.8 ± 4.7 points	14 people	20 people	41.2%
Wingers	36.5 ± 4.6 points	30 people	34 people	46.9%
Back players	37.4 ± 7.5 points	28 people	32 people	46.7%
Pivots	36.8 ± 4.7 points	12 people	18 people	40.0%
Goalkeepers	36.7 ± 7.4 points	12 people	20 people	37.5%

**Table 6:** Reaction time results per position.

	Average	Passed	Failed	Success %
Goalkeepers	0.6 ± 0.1 s	30 people	2 people	93.8%
Center backs	0.6 ± 0.1 s	28 people	6 people	82.4%
Wingers	0.6 ± 0.1 s	48 people	16 people	75.0%
Back players	0.7 ± 0.1 s	40 people	20 people	66.7%
Pivots	0.7 ± 0.1 s	18 people	12 people	60.0%

**Table 7:** Dismiss results per position.

	Average	Passed	Failed	Success %
Pivots	4.9 ± 3.0 points	28 people	2 people	93.3%
Wingers	5.2 ± 2.4 points	64 people	0 people	100.0%
Goalkeepers	5.4 ± 3.2 points	32 people	0 people	100.0%
Back players	5.8 ± 4.0 points	54 people	6 people	90.0%
Center backs	6.4 ± 3.4 points	32 people	2 people	94.1%

**Table 8:** Neurocognitive performance per position and sex.

N = 220 People		Women (N = 104 People)				Men (N = 116 People)			
Competency	Position	Average	Passed	Failed	%	Average	Passed	Failed	%
Verbal memory	Wingers	86.0 ± 9.1%	26 people	4 people	86.7%	83.8 ± 11.1%	20 people	14 people	58.8%
	Back players	85.6 ± 9.7%	24 people	6 people	80.0%	81.9 ± 10.8%	14 people	16 people	46.7%
	Pivots	84.0 ± 10.2%	10 people	6 people	62.5%	84.0 ± 8.3%	10 people	4 people	71.4%
	Center backs	73.6 ± 15.8%	6 people	8 people	42.9%	83.3 ± 11.5%	14 people	6 people	70.0%
	Goalkeepers	82.9 ± 8.2%	8 people	6 people	57.1%	80.3 ± 9.8%	10 people	8 people	55.6%
Visual memory	Wingers	75.7 ± 10.9%	26 people	4 people	86.7%	74.8 ± 9.3%	32 people	2 people	94.1%
	Back players	74.6 ± 11.9%	24 people	6 people	80.0%	72.6 ± 8.9%	24 people	6 people	80.0%
	Pivots	71.8 ± 17.3%	10 people	6 people	62.5%	72.3 ± 11.2%	10 people	4 people	71.4%
	Center backs	70.6 ± 9.6%	12 people	2 people	85.7%	70.6 ± 8.2%	16 people	4 people	80.0%
	Goalkeepers	73.0 ± 11.2%	12 people	2 people	85.7%	70.7 ± 17.7%	12 people	6 people	66.7%
Visual–motor speed	Wingers	36.2 ± 4.8 p	14 people	16 people	46.7%	36.9 ± 4.8 p	16 people	18 people	47.1%
	Back players	36.3 ± 6.7 p	14 people	16 people	46.7%	38.1 ± 7.6 p	14 people	16 people	46.7%
	Pivots	37.6 ± 5.5 p	8 people	8 people	50.0%	35.9 ± 3.9 p	4 people	10 people	28.6%
	Center backs	38.1 ± 6.4 p	6 people	8 people	42.9%	37.7 ± 3.4 p	8 people	12 people	40.0%
	Goalkeepers	37.6 ± 8.7 p	6 people	8 people	42.9%	35.9 ± 6.6 p	6 people	6 people	33.3%
Reaction time	Wingers	0.6 ± 0.1 s	22 people	8 people	73.3%	0.6 ± 0.1 s	26 people	8 people	76.5%
	Back players	0.7 ± 0.1 s	18 people	12 people	60.0%	0.6 ± 0.1 s	22 people	8 people	73.3%
	Pivots	0.7 ± 0.1 s	8 people	8 people	50.0%	0.6 ± 0.1 s	10 people	4 people	71.4%
	Center backs	0.6 ± 0.1 s	12 people	2 people	85.7%	0.6 ± 0.1 s	16 people	4 people	80.0%
	Goalkeepers	0.6 ± 0.1 s	12 people	2 people	85.7%	0.6 ± 0.1 s	18 people	0 people	100.0%
Dismiss	Wingers	5.3 ± 2.3 p	30 people	0 people	100.0%	5.0 ± 2.6 p	34 people	0 people	100.0%
	Back players	5.8 ± 3.7 p	28 people	2 people	93.3%	5.7 ± 4.5 p	26 people	4 people	86.7%
	Pivots	5.6 ± 3.6 p	14 people	2 people	87.5%	4.0 ± 2.2 p	14 people	0 people	100.0%
	Center backs	5.0 ± 2.8 p	14 people	0 people	100.0%	7.4 ± 3.6 p	18 people	2 people	90.0%
	Goalkeepers	4.4 ± 2.9 p	14 people	0 people	100.0%	6.2 ± 3.4 p	18 people	0 people	100.0%

**Table 9:** Stability test results per position.

	Two-Leg Stability	One-Leg Stability D	One-Leg Stability ND	Symmetry Index
Wingers	2.4 ± 0.8 point	2.4 ± 0.7 point	2.4 ± 0.7 point	86.8 ± 9.2%
Back players	2.5 ± 0.7 point	2.7 ± 0.8 point	2.5 ± 0.8 point	88.2 ± 8.2%
Pivots	2.6 ± 1.0 point	2.8 ± 0.8 point	2.5 ± 0.7 point	87.1 ± 10.7%
Goalkeepers	2.7 ± 0.9 point	3.0 ± 0.9 point	2.7 ± 0.7 point	85.5 ± 14.9%
Center backs	2.8 ± 0.6 point	2.6 ± 0.6 point	2.7 ± 0.6 point	88.7 ± 8.3%

**Table 10:** Plyometrics and speed results per position.

	Plyometric Jump Height	Plyometric Jump Ground Contact Time	Quick Feet
Wingers	30.8 ± 7.7 cm	163.8 ± 30.6 ms	8.0 ± 0.8 s
Back players	30.6 ± 7.0 cm	170.1 ± 29.5 ms	8.1 ± 1.3 s
Pivots	30.5 ± 9.8 cm	174.1 ± 32.0 ms	7.7 ± 0.7 s
Center backs	31.4 ± 7.6 cm	186.4 ± 32.4 ms	8.0 ± 0.8 s
Goalkeepers	28.5 ± 6.0 cm	183.3 ± 30.2 ms	8.1 ± 0.8 s

**Table 11:** Agility and Parkour system results per position.

	Parkour D	Parkour ND	Symmetry Index
Wingers	6.7 ± 0.6 s	6.6 ± 0.5 s	94.9 ± 3.9%
Center backs	6.7 ± 0.8 s	6.6 ± 0.9 s	93.7 ± 5.4%
Back players	6.8 ± 1.2 s	6.8 ± 1.6 s	94.7 ± 4.9%
Pivots	6.9 ± 0.6 s	6.7 ± 0.7 s	95.1 ± 5.4%
Goalkeepers	7.3 ± 1.1 s	7.2 ± 1.2 s	93.6 ± 6.0%

**Table 12:** Neuromuscular performance per position and sex.

N = 220 People		Women (N = 104 People)				Men (N = 116 People)			
Competency	Position	Average	Passed	Failed	%	Average	Passed	Failed	%
Two-leg stability	Wingers	1.8 ± 0.6 points	30 people	0 person	100%	2.9 ± 0.5 points	34 people	0 person	100%
	Back players	2.1 ± 0.5 points	30 people	0 person	100%	2.9 ± 0.7 points	26 people	4 people	86.7%
	Pivots	2.0 ± 0.7 points	16 people	0 person	100%	3.3 ± 0.9 points	10 people	4 people	71.4%
	Center backs	2.5 ± 0.5 points	12 people	2 person	85.7%	2.9 ± 0.5 points	18 people	2 person	90%
	Goalkeepers	2.1 ± 0.5 points	14 people	0 person	100%	3.2 ± 0.9 points	14 people	4 people	77.8%
One-leg stability D	Wingers	1.9 ± 0.5 points	30 people	0 person	100%	2.7 ± 0.6 points	32 people	2 person	94.1%
	Back players	2.2 ± 0.5 points	26 people	4 people	86.7%	3.0 ± 0.9 points	26 people	4 people	86.7%
	Pivots	2.3 ± 0.6 points	14 people	2 person	87.5%	3.3 ± 0.7 points	10 people	4 people	71.4%
	Center backs	2.3 ± 0.5 points	12 people	2 person	85.7%	2.8 ± 0.7 points	16 people	4 people	80%
	Goalkeepers	2.5 ± 1.0 points	12 people	2 person	85.7%	3.3 ± 0.7 points	14 people	4 people	77.8%
One-leg stability ND	Wingers	2.0 ± 0.5 points	30 people	0 person	100%	2.8 ± 0.6 points	30 people	4 people	88.2%
	Back players	2.3 ± 0.5 points	26 people	4 people	86.7%	3.1 ± 0.8 points	28 people	2 person	93.3%
	Pivots	2.1 ± 0.5 points	16 people	0 person	100%	3.1± 0.5 points	10 people	4 people	71.4%
	Center backs	2.5 ± 0.4 points	12 people	2 person	85.7%	2.9 ± 0.6 points	18 people	2 person	90%
	Goalkeepers	2.2 ± 0.5 points	14 people	0 person	100%	3.0 ± 0.8 points	16 people	2 person	88.9%
Plyometric height	Wingers	27.0 ± 4.0 cm	30 people	0 person	100%	34.1 ± 8.7 cm	34 people	0 person	100%
	Back players	28.6 ± 6.8 cm	26 people	4 people	86.7%	32.6 ± 6.9 cm	26 people	4 people	86.7%
	Pivots	26.6 ± 6.6 cm	16 people	0 person	100%	34.9 ± 11.4 cm	14 people	0 person	100%
	Center backs	28.6 ± 5.7 cm	14 people	0 person	100%	32.6 ± 8.6 cm	16 people	4 people	80%
	Goalkeepers	26.8 ± 7.1 cm	10 people	4 people	71.4%	29.8 ± 5.0 cm	14 people	4 people	77.8%
Plyometric ground contact time	Wingers	153.4 ± 18.3 ms	30 people	0 person	100%	173.1 ± 36.5 ms	34 people	0 person	100%
	Back players	158.3 ± 19.2 ms	26 people	4 people	86.7%	181.9 ± 33.7 ms	26 people	4 people	86.7%
	Pivots	164.9 ± 23.9 ms	16 people	0 person	100%	184.6 ± 38.1 ms	14 people	0 person	100%
	Center backs	153.9 ± 23.9 ms	14 people	0 person	100%	196.7 ± 34.7 ms	16 people	4 people	80%
	Goalkeepers	179.7 ± 22.3 ms	10 people	4 people	71.4%	186.0 ± 36.3 ms	14 people	4 people	77.8%
Parkour D	Wingers	7.0 ± 0.5 s	30 people	0 person	100%	6.5 ± 0.4 s	34 people	8 people	76.5%
	Back players	6.6 ± 0.6 s	30 people	0 person	100%	6.9 ± 1.7 s	22 people	8 people	73.3%
	Pivots	6.9 ± 0.4 s	16 people	0 person	100%	6.9 ± 0.7 s	8 people	6 people	57.1%
	Center backs	6.9 ± 0.5 s	14 people	0 person	100%	6.5 ± 0.8 s	14 people	6 people	70%
	Goalkeepers	8.1 ± 1.0 s	12 people	2 person	85.7%	6.8 ± 0.8 s	8 people	10 people	44.4%
Parkour ND	Wingers	6.7 ± 0.6 s	30 people	0 person	100%	6.5 ± 0.6 s	34 people	8 people	76.5%
	Back players	6.8 ± 0.5 s	30 people	0 person	100%	7.0 ± 2.3 s	18 people	12 people	60%
	Pivots	6.9 ± 0.8 s	16 people	0 person	100%	6.5 ± 0.5 s	12 people	2 person	85.7%
	Center backs	7.0 ± 0.5 s	14 people	0 person	100%	6.5 ± 1.0 s	16 people	4 people	80%
	Goalkeepers	7.7 ± 0.6 s	14 people	0 people	100%	6.8 ± 1.3 s	12 people	6 people	55.6%
Quick feet	Wingers	8.1 ± 0.9 s	30 people	0 person	100%	7.9 ± 0.7 s	34 people	0 person	100%
	Back players	8.0 ± 1.0 s	30 people	0 person	100%	8.2 ± 1.6 s	28 people	2 person	93.3%
	Pivots	8.1 ± 0.9 s	16 people	0 person	100%	8.0 ± 0.8 s	14 people	0 person	100%
	Center backs	7.4 ± 0.5 s	14 people	0 person	100%	7.8 ± 0.7 s	20 people	0 person	100%
	Goalkeepers	8.4 ± 0.4 s	14 people	0 people	100%	8.0 ± 1.0 s	18 people	0 person	100%

## Data Availability

The datasets generated and/or analyzed during the current study are not publicly available due to ethical and privacy restrictions but are available from the corresponding author on reasonable request.

## Supplementary Materials

The following supporting information can be downloaded at: https://www.mdpi.com/article/10.3390/sports14050185/s1, Figure S1: Stabilometer of BIA system (own picture); Figure S2: Center of Pressure displayed on the screen (own picture); Figure S3: Parkour subtest (own picture); Figure S4: Quick–feet subtest (own picture); Figure S5: The composite scores and their subtests.
