# Supplementary material for: Neuromuscular and Neurocognitive Performance Associated with ACL Injury Risk in Youth Handball Players: A Prospective Cohort Study
Source: Sports (Basel). 2026 May 6;14(5):185. doi: 10.3390/sports14050185 (PMC13210643; doi:10.3390/sports14050185)
Supplement: Supplementary file 1 [file sports-14-00185-s001.zip › sports-4269742-supplementary.pdf]

Back in Action testing battery

General Protocol For every subtest, one practice trial was provided to ensure task familiarity before the recorded measurements began.

1. Stabilometry: Participants stood facing a monitor for the assessment of double-leg stability. A visual target was displayed on the screen, with a circular cursor representing the displacement of the Center of Pressure (CoP). The athletes were instructed to maintain the cursor in the center of the target with minimal oscillation for 20 seconds. 3 attempts, 60-second rest period -automatically signaled by the system between attempts. Single-leg stabilometry followed the same protocol; however, participants were required to maintain balance without the non-standing limb touching the board, the ground, or the athlete's own body. The number of attempts, test duration, and rest intervals were identical to the bipedal measurement.

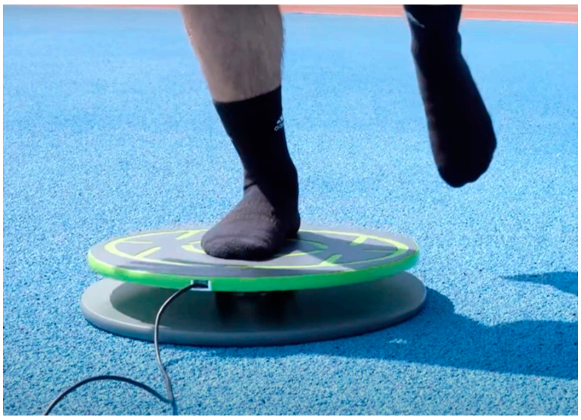

Figure S1: Stabilometer of BIA system (own picture)

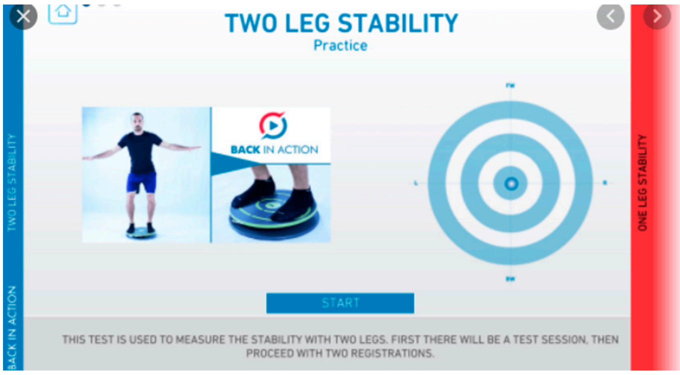

Figure S2: Center of Pressure displayed on the screen (own picture)

2. Countermovement Jumps (CMJ): CMJ performance was measured using a sensor secured to the waist with an elastic belt, with hands placed on the hips to eliminate arm swing. For the two-leg CMJ, athletes jumped vertically with maximal effort and landed in a stable, bilateral squat position. The sensor recorded jump height and ground reaction forces (GRF). A total of 6 repetitions were performed with 15-second automated rest intervals. For single-leg CMJ, the sensor was positioned over the jumping limb. Athletes performed a single-leg take-off followed by a bilateral landing. This subtest consisted of 3 repetitions per leg with 15-second rest periods.

3. Plyometric Jumps: The experimental setup was the same, like the two-leg CMJ, though athletes were permitted to use their arms. The protocol involved an initial maximal vertical jump, followed immediately by four consecutive, dynamic "reactive" jumps upon ground contact, primarily utilizing an ankle strategy. This test aimed to assess leg stiffness during landing phases. Four repetitions were performed with 15-second rest intervals.

4. Parkour Obstacle Course The obstacle course was constructed using two distinct colors: blue and red. The blue bars formed the central frame, while the red bars functioned as lateral hurdles. The sequence consisted of three jumps over the red bars (forward, backward, forward) followed by one lateral jump over the blue bar. This task was performed continuously on one leg as quickly as possible. Three repetitions were completed per side, with 1-minute rest periods.

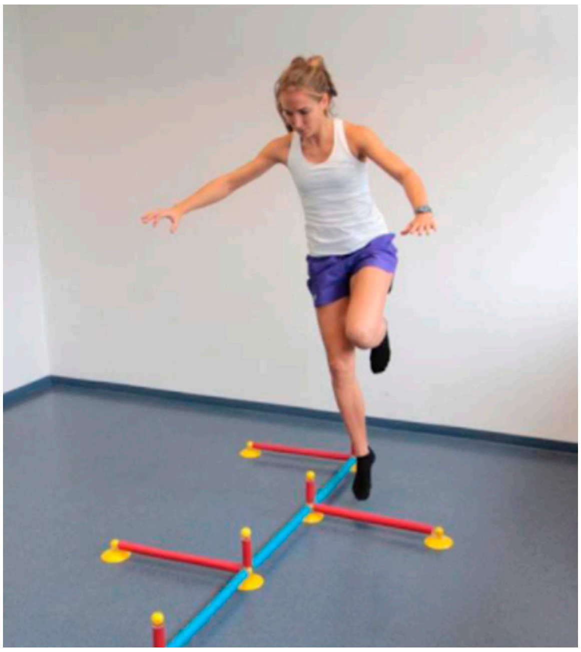

Figure S3: Parkour subtest (own picture)

5. Quick-feet Test Athletes performed a rapid stepping task within rectangles defined by the bars. Starting from a wide stance outside the frame, the athlete performed in-and-out stepping movements continuously. One complete cycle (stepping in and out with both feet) was counted as one repetition. Participants were required to complete 15 cycles at maximal speed. The test consisted of 3 repetitions with 1-minute rest periods.

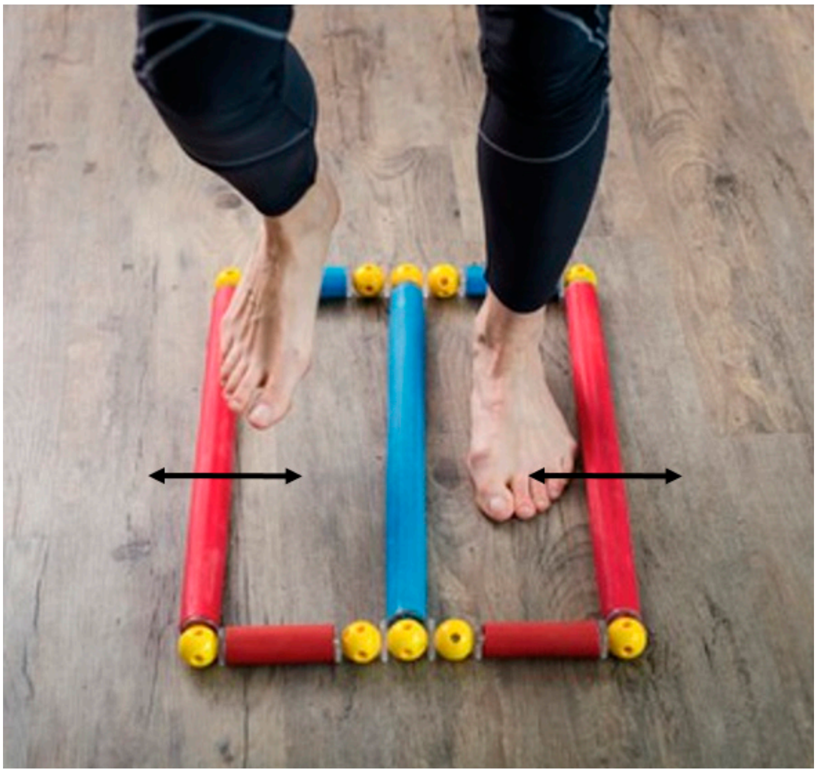

Figure S4: Quick – feet subtest (own picture)

ImPACT Neurocognitive testing system

The assessment followed the standardized ImPACT protocol. To mitigate potential learning effects and task adaptation, participants were prohibited from performing any warm-up or practice sessions prior to the administration of the test.

Task Structure and Randomization Each task type consisted of three repetitions; however, to ensure the validity of the results, the software employed automated randomization. This involved dynamic changes in the spatial positioning of stimuli (e.g., symbols and numbers), thereby preventing pattern recognition and ensuring that each attempt remained a novel cognitive challenge.

Scoring and Evaluation The determination of whether an athlete met the "pass" criteria was automatically generated by the software, based on the specific thresholds detailed in *Picture 4*. The system calculates several Composite Scores, each of which is synthesized from multiple subtests. These scores are aggregated using the system’s proprietary algorithm to provide a comprehensive profile of the athlete's neurocognitive status (including verbal/visual memory, processing speed, and reaction time).

**Composite score Verbal Memory calculation**	Word memory total percent correct
	Symbol match (total correct hidden) /9*100
	Three Letters percent total letters correct
	Total divided by 3
**Composite score Visual Memory calculation**	Design mermory total correct percent
	X's and O's (total correct memory)/12*100
	Total divided by 2
**Composite score Visual-Motor Speed**	X's and O's total correct (interference)/4
	Three letters average counted correctly*3
	Total divided by 2
**Composite Reaction Time**	X's and O's Avg. correct RT(interference)
	Symbol match Avg. correct RT (visible)/3
	Color match Avg. correct RT
	Total divided by 3
**Composite Dismiss**	X's and O's Total incorrect (interference)
	Color match total comissions
	Total score (summarize)

	Perfect	Optimal
Word Memory		
Hits (immediate)	12	11-12
Correct Distractors (immed	12	11-12
Learning Perfect Correct	100%	90%
Hits (Delay)	12	11-12
Correct Distractors (delay)	12	11-12
Delayed Memory pct Correc	100%	90%
Total Percent correct	100%	90%

	Perfect	Optimal
Design Memory		
Hits (immediate)	12	9-12
Correct Distractors (immed	12	9-12
Learning Perfect Correct	100%	80%
Hits (Delay)	12	9-12
Correct Distractors (delay)	12	9-12
Delayed Memory pct Correc	100%	80%
Total Percent correct	100%	80%

	Perfect	Optimal
X's and O's		
Total Correct (memory)	12	9-12
Total Correct (interference)	n/a	120+
Avg. Correct RT (interferenc	n/a	< 0.50
Avg. Incorrect (interference)	0	0-10
Avg. Incorrect RT (interferer	n/a	< 0.50

	Perfect	Optimal
Symbol Match		
Total Correct (visible)	27	26-27
Avg. Correct RT (visible)	n/a	< 1.50
Total Correct (hidden)	9	7-9
Avg. Correct RT (hidden)	n/a	n/a

	Perfect	Optimal
Color Match		
Total Correct	9	8-9
Avg. Correct RT	n/a	< .75
Total Commissions	0	0-1
Avg. Commissions RT	n/a	n/a

	Perfect	Optimal
Three Letters		
Total Sequence Correct	5	4-5
Total Letters Correct	15	14-15
Pct. Of Total Letters Correc	100%	95%
Avg. time first click	n/a	n/a
Avg. counted	25.0	> 15.0
Avg. counted correctly	25.0	> 15.0

Figure S5: The composite scores and their subtests
